# Errata

**DOI:** 10.36660/abc.20200730

**Published:** 2020-07-28

**Edição de Abril de 2020, vol. 114 (4), págs. 732-735**

Na Comunicação Breve “Ações Transmurais Inotrópicas e Antiarrítmicas da Ranolazina em um Modelo Celular da Síndrome do QT Longo Tipo 3”, com número de DOI: https://doi.org/10.36660/abc.20190220, publicado no periódico Arquivos Brasileiros de Cardiologia, 114(4):732-735, na página 732: considerar correta a grafia Danilo Roman-Campos para o nome do autor Danilo Roman Campos.

**Edição de Maio de 2020, vol. 114 (5), págs. 849-942**

No Posicionamento “Posicionamento da Sociedade Brasileira de Cardiologia para Gravidez e Planejamento Familiar na Mulher Portadora de Cardiopatia – 2020”, com número de DOI: https://doi.org/10.36660/abc.20200406, publicado no periódico Arquivos Brasileiros de Cardiologia, 114 (5): 849-942, na página 851, no conflito de interesses do Dr. Fernando Souza Nani, no item ”Foi palestrante em eventos ou atividades patrocinadas pela indústria relacionados ao posicionamento em questão”, considerar correta a empresa CSL Behring ao invés de Boehringer.

**Edição de Março de 2020, vol. 114 (3), pág. 582**

No Posicionamento “Posicionamento Brasileiro sobre Hipertensão Arterial Resistente – 2020”, com número de DOI: https://doi.org/10.36660/abc.20200198, publicado no periódico Arquivos Brasileiros de Cardiologia, 114(3): 576-596, na página 582: na figura 1 da versão português, onde é mencionado “hipertensão segundária”, o correto é “Hipertensão arterial pseudorresistente”. Na versão em inglês, onde “anormal” é mencionado, lado direito da figura 1, o correto é “normal”. Na versão em inglês, onde “abnormal” é mencionado, lado direito da figura 1, o correto é “normal”.

**Ahead of Print**

No artigo original publicado em ahead of print com o título “Avaliação do Tempo de Condução Atrioventricular Dinâmica para Acoplamento ao Intervalo RR em Atletas e Indivíduos Sedentários”, com número de DOI: https://doi.org/10.36660/abc.20190281, publicado no periódico Arquivos Brasileiros de Cardiologia, considerar correto o título “Avaliação da Dinâmica do Acoplamento da Condução Atrioventricular à Variação dos Intervalos RR em Atletas e Indivíduos Sedentários”.

