# Supplementary material for: Errata
Source: Arq Bras Cardiol. 2020 Jul 28;115(1):148. [Article in Portuguese] doi: 10.36660/abc.20200730 (PMC8384310; doi:10.36660/abc.20200730)
Supplement: Translation

# Translation: Erratum

**April 2020 Issue, vol. 114 (4), pages 732-735**

In the Brief Communication “Inotropic and Antiarrhythmic Transmural Actions of Ranolazine in a Cellular Model of Type 3 Long QT Syndrome”, with DOI number: https://doi.org/10.36660/abc.20190220, published in the periodical Arquivos Brasileiros de Cardiologia, 114(4):732-735, on page 732: consider Danilo Roman-Campos as the correct form for the name of the author Danilo Roman Campos.

**May 2020 Issue, vol. 114 (5), pages 849-942**

In the Statement “Brazilian Cardiology Society Statement for Management of Pregnancy and Family Planning in Women with Heart Disease – 2020”, with DOI number: https://doi.org/10.36660/abc.20200406, published in the periodical Arquivos Brasileiros de Cardiologia, 114(5): 849-942, on page 851, in the conflict of interests of Dr. Fernando Souza Nani, in the item “Spoke at events or activities sponsored by industry related to this statement”, consider the company CSL Behring to be correct instead of Boehringer.

**March 2020 Issue, vol. 114 (3), page 582**

In the Statement “Posicionamento Brasileiro sobre Hipertensão Arterial Resistente – 2020” with DOI number: https://doi.org/10.36660/abc.20200198, published in the periodical Arquivos Brasileiros de Cardiologia, 114(3): 576-596, on page 582: in the figure 1 of the Portuguese version, where “hipertensão segundária” is mentioned, the correct is “Hipertensão arterial pseudorresistente”. In the English version, where “abnormal” is mentioned, right side of the figure 1, the correct is “normal”.

**Ahead of Print**

In the original article published in ahead of print with the title “Avaliação do Tempo de Condução Atrioventricular Dinâmica para Acoplamento ao Intervalo RR em Atletas e Indivíduos Sedentários”, with DOI number: https://doi.org/10.36660/abc.20190281, published in the periodical Arquivos Brasileiros de Cardiologia, consider the title correct: “Avaliação da Dinâmica do Acoplamento da Condução Atrioventricular à Variação dos Intervalos RR em Atletas e Indivíduos Sedentários”.
